# Developmental predictors of young adult borderline personality disorder: a prospective, longitudinal study of females with and without childhood ADHD

**DOI:** 10.1186/s12888-023-04515-3

**Published:** 2023-02-15

**Authors:** Sinclaire M. O’Grady, Stephen P. Hinshaw

**Affiliations:** 1 Department of Psychology, University of California, Berkeley, 2121 Berkeley Way West, Berkeley, CA 94720-1650 USA; 2 Department of Psychiatry and Behavioral Sciences, University of California, 675 18th Street, San Francisco, San Francisco, CA 94107 USA

**Keywords:** Borderline personality disorder, Attention-deficit hyperactivity disorder, Risk factors, Longitudinal studies, Adverse childhood experiences

## Abstract

**Background:**

Research on the precursors of borderline personality disorder (BPD) reveals numerous child and adolescent risk factors, with impulsivity and trauma among the most salient. Yet few prospective longitudinal studies have examined pathways to BPD, particularly with inclusion of multiple risk domains.

**Methods:**

We examined theory-informed predictors of young-adult BPD (a) diagnosis and (b) dimensional features from childhood and late adolescence via a diverse (47% non-white) sample of females with (*n* = 140) and without (*n* = 88) carefully diagnosed childhood attention-deficit hyperactivity disorder (ADHD).

**Results:**

After adjustment for key covariates, low levels of objectively measured executive functioning in childhood predicted young adult BPD diagnostic status, as did a cumulative history of childhood adverse experiences/trauma. Additionally, both childhood hyperactivity/impulsivity and childhood adverse experiences/trauma predicted young adult BPD dimensional features. Regarding late-adolescent predictors, no significant predictors emerged regarding BPD diagnosis, but internalizing and externalizing symptoms were each significant predictors of BPD dimensional features. Exploratory moderator analyses revealed that predictions to BPD dimensional features from low executive functioning were heightened in the presence of low socioeconomic status.

**Conclusions:**

Given our sample size, caution is needed when drawing implications. Possible future directions include focus on preventive interventions in populations with enhanced risk for BPD, particularly those focused on improving executive functioning skills and reducing risk for trauma (and its manifestations). Replication is required, as are sensitive measures of early emotional invalidation and extensions to male samples.

## Background

Borderline personality disorder (BPD) is a persistent and highly impairing condition characterized by intense and pervasive dysregulation of emotion, behavior, and cognition, and a pattern of highly unstable interpersonal relationships [1]. Individuals with BPD are at extremely high risk for suicide: Up to 10% of individuals with BPD die by suicide each year, 50 times higher than the rate in the general population [2, 3]. BPD is also associated with significant personal (i.e., severe psychosocial impairment) and economic/public health (i.e., high rates of underemployment and increased disability) consequences. In fact, although individuals with BPD comprise 1–2% of the general population [4], they have extremely high rates of health service use, representing up to 20% of individuals receiving inpatient psychiatric treatment and 10% receiving outpatient psychiatric care [5]. Evidence-based psychological treatments (e.g., Dialectical Behavior Therapy [DBT]) exist for BPD, with strong evidence for efficacy [6]. Yet they are resource-intensive, with limited availability of expert providers.

Given the high morbidity, mortality, and public health consequences of BPD, an urgent need exists to identify individuals at risk for its development. Relevant research is accumulating. A leading model is Linehan’s Biosocial Theory, which proposes that BPD emerges from transactions between (a) biological vulnerabilities linked with both impulsivity and emotional sensitivity and (b) specific environmental influences such as social invalidation, adversity, or trauma [1, 7]. Across development, such combinations give rise to increasingly extreme emotional, behavioral, cognitive, and interpersonal dysregulation for vulnerable individuals. Although empirical research has generated numerous potential risk factors for BPD [8, 9], relatively few studies have examined longitudinal pathways to BPD. Identification of such would help strengthen theoretical approaches to the development of BPD. In particular, few studies have examined childhood risk factors for BPD in prospective designs [10]. Indeed, a 2016 systematic review of risk factors for BPD revealed that risk factors were assessed mainly during early adolescence (*M*_age_ = 13 years), highlighting the need for further investigation of childhood variables and processes [9]. Such studies could further inform leading theories [1, 11] regarding heightened periods of risk (and areas for intervention). In the present investigation, we examine both child and late-adolescent risk factors for later BPD.

### Developmental risk factors for BPD

#### ADHD symptoms

Impulsivity is a key feature in both the development and presentation of BPD [1]. It is also a core symptom of attention-deficit hyperactivity disorder (ADHD). In fact, several studies have reported high comorbidity between BPD and ADHD [12, 13]. In a large national study of 34,000 adults in the United States, among adults with ADHD the lifetime comorbidity with BPD was 33.7% [14]. Comorbid ADHD and BPD is a particularly impairing combination [12]. As for childhood ADHD in relation to adult BPD, most prior research has involved retrospective reports of symptoms. Here, 50–60% of adults with BPD endorsed high levels of ADHD symptoms in childhood [15]. Philipsen et al. [16] reported estimates of ADHD among adult women with BPD to be especially high during childhood (41.5%) as compared to adulthood (16.1%). Severity of childhood ADHD symptoms is also associated with higher frequency of personality disorder diagnoses, including BPD, by adulthood [17].

Hypothesized mechanisms linking ADHD and BPD focus on the interaction, across development, between early impulsivity (a highly heritable trait) and emotion dysregulation, the combination of which, in turn, is shaped by adverse socialization processes (e.g., maltreatment, family reinforcement of emotional lability) [11]. Proposed neurobiological mechanisms focus on dysfunction in the prefrontal cortex, which is also implicated in emotion regulatory capacities [11, 18]. Essentially, early ADHD is hypothesized to confer risk for later BPD because of its associated behavioral dysregulation, promoting environmental reinforcement of maladaptive behaviors and often leading to a pervasive and difficult-to-treat cycle of dysregulation.

As noted above, however, few prospective studies have examined the link between childhood ADHD and later BPD. Notable exceptions (see [19–21]) reveal that childhood ADHD predicts personality disorders, including BPD, later in life. In a follow-up investigation of “hyperactive” children in young adulthood, Fischer et al. [22] found that 14% of hyperactive participants met criteria for BPD compared to 3% of their comparison group. Miller et al. [13] found that among a group with childhood ADHD, 13.5% were diagnosed with BPD in adolescence compared to 1.2% in their comparison group. Furthermore, in the Pittsburgh Girls Study, Stepp et al. [18] found that high levels of ADHD symptoms during childhood predicted BPD in adolescence (see also [23] for parallel findings in males). Using latent-class analysis, Thatcher et al. [24] found that the presence of ADHD symptoms in adolescents, along with substance use disorders, predicted more severe BPD symptoms at young-adult follow-up.

The majority of relevant research has focused on the categorical diagnosis of ADHD rather than the core ADHD dimensions of (a) hyperactivity/impulsivity and (b) inattention. Exceptions include Carlson et al. [25], who reported data from a prospective, longitudinal study. Teacher-rated severity of both attentional disturbance and behavioral instability (including impulsivity) at age 12 was predictive of adult BPD. This finding was recently replicated by Beeney et al. [8] in a prospective study of females. Here, parent- and child-reported severity of hyperactivity, impulsivity, and inattention at ages 14–15 predicted BPD at ages 16–18. In a prospective study in twins, maternal- and teacher-rated symptoms of impulsivity at age 5 were related to borderline symptoms at age 12 [26]. Finally, a recent national, prospective longitudinal study of twins in Sweden found that the association between childhood ADHD symptoms, assessed at age 9 or 12, and adult BPD was primarily driven by impulsivity as opposed to inattention or hyperactivity [27].

Some adult research has examined presentations (or “types”) of ADHD as related to BPD. Among adults with ADHD, one study reported a higher prevalence rate of comorbid BPD in the Combined presentation of ADHD (ADHD-C; 24%), for which hyperactivity/impulsivity and inattention are key components, compared to comorbid BPD and the Inattentive presentation (ADHD-I; 10%) [28]. Using latent class analysis with adult females, [29] found that one pathway to adult BPD emanated from a childhood profile with at least low/moderate levels of hyperactive/impulsive symptoms but low levels of inattentive symptoms.

Finally, considered either categorically or dimensionally, ADHD is clearly linked with increased risk for self-harm (a common feature of BPD), including suicidal behavior and nonsuicidal self-injury (NSSI) (see [30] for a recent review). Notably, females with ADHD, especially those with high levels of impulsivity as characterized by the ADHD-C presentation, are markedly at risk for attempted suicide and moderate-to-severe NSSI [31]. Mechanisms linking ADHD with later self-harm include internalizing and externalizing symptoms, as well as peer victimization and peer rejection [30]. Risk for suicidality is greatly increased when females with ADHD have histories of childhood maltreatment [32].

#### Executive functioning (EF)

EF includes goal-oriented cognitive processes such as planning, inhibition, organization, set shifting, working memory, and problem solving. EF deficits have frequently been linked to both ADHD and BPD. For a review of ADHD and EF see Brown [33]; for a review of BPD and EF, see Garcia-Villamisar et al. [34]. In general, individuals with BPD show greater EF deficits than typically developing controls [35]. Individuals with BPD have demonstrated EF deficits in the domains of planning [36, 37], working memory [38], response inhibition and problem solving [39], and motor inhibition [40]. Additionally, a meta-analysis revealed that BPD samples with higher rates of comorbid psychopathology performed worse on EF tasks compared to samples with lower rates of comorbidity [41]. However, few prospective longitudinal studies have examined childhood EF as predictive of later BPD, with the notable exception of Belsky et al. [26], who found that a composite measure of EF deficits at age 5 was related to BPD symptoms at age 12. Longitudinal investigation of global measures of EF and relations to BPD are critically needed.

#### Early internalizing and externalizing symptoms

BPD is commonly comorbid with a variety of other psychological disorders, both internalizing and externalizing in nature [42]. In a systematic review of risk factors for BPD from longitudinal research, Stepp et al. [43] found that 16 of 19 studies examining internalizing and externalizing psychopathology yielded predictions to later BPD [9]. That is, dimensions of internalizing (depression) and externalizing (substance use disorder) behaviors in adolescence were associated with subsequent adult BPD symptoms. Indeed, existing literature theorizes that adolescence is a sensitive period for the development of personality disorders—and that personality disorders are preceded by internalizing and externalizing symptoms, not the other way around [44]. Additionally, some evidence indicates that internalizing and externalizing symptoms in childhood are also related to later BPD symptoms (Belsky et al., [26]; Geselowitz et al., [10] but see Burke [23] & Stepp, [18], for negative results). Hypothesized mechanisms include emotion dysregulation, which characterizes both internalizing and externalizing psychopathology [11]. In short, greater understanding of the contribution and developmental timing of internalizing and externalizing symptoms has the potential to inform early interventions to prevent the development of clinically significant BPD symptoms.

#### Adverse childhood experiences/trauma

Consistent with Linehan’s Biosocial Model, a large body of research has linked a history of environmental invalidation and adversity—and at its extreme, trauma—to the development of BPD [1]. A history of physical abuse, sexual abuse, and neglect in childhood has long been linked with BPD [9]. For key prospective longitudinal investigations, see Johnson et al. [45] and Widom et al. [46]. Having a parent with psychopathology (including depression and substance use problems) has consistently been found to be a family-related risk factor for BPD [9, 43]. Empirical research on the role of parenting and parent–child transactions has been limited [47], even though key theories posit that transactions between a child’s biological sensitivity and adverse environments (including family factors and parenting) both lead to and maintain BPD symptomology [1] as well self-harmful behaviors in early adulthood [48]. High levels of parental depression and parental stress have been linked to BPD [49], as well as escalation of negative affect and behaviors in mother-daughter conflict situations [47]. In a prospective, longitudinal study of twins followed from age 5 to 12, children who were physically maltreated or exposed to high maternal negative expressed emotion developed high levels of BPD characteristics [26], replicating other prospective studies [25, 50, 51]. Still, at least one study revealed that maternal parenting stress in adolescence was not related to adolescent BPD symptom severity [52]. More research is needed related to transactions between parents and their offspring in terms of the development of BPD [53].

### Present study and hypotheses

In sum, numerous risk factors for BPD have been posited, including ADHD symptom dimensions, low executive functioning, early internalizing and externalizing psychopathology, and childhood adversity and trauma. Yet with the clear exception of Beeney et al. [8], little research has examined such factors simultaneously, limiting current understanding of the independent or combined contributions of such variables. Also, many studies examine delimited developmental periods (e.g., childhood to adolescence, adolescence to young adulthood). Finally, there is a dearth of research examining the core ADHD dimensions of hyperactivity/impulsivity and inattention as related to risk for later BPD (for a review, see Beauchaine et al. [11]).

We leverage a sample of females with childhood-diagnosed ADHD and a matched comparison sample followed prospectively from childhood through young adulthood. Consistent with Linehan’s Biosocial Model, recent developmental models of females with ADHD [11], and extant literature, we hypothesize that both childhood and adolescent (a) hyperactivity/impulsivity and (b) adversity/trauma will emerge as significant risk factors for BPD after adjusting for demographic covariates as well as additional evidence-based risk factors. We also predict that, by late adolescence, internalizing and externalizing symptoms will be significant predictors of young adult BPD. An exploratory aim is to examine if childhood socioeconomic status (SES) moderates associations between predictors of interest and later BPD. We aim to add to the literature on developmental risk factors for BPD to inform existing models of BPD development and prevention approaches.

## Method

### Procedure and participants

The current data were drawn from an ongoing prospective, longitudinal study of females with and without carefully diagnosed childhood ADHD (see Hinshaw, [54] for more complete details). This study was approved by the Committee for the Protection of Human Subjects (CPHS) at the University of California, Berkeley. Participants were initially recruited across a metropolitan area from schools, mental health centers, pediatric practices, and through advertisements to participate in research-based, 5-week summer day camps between 1997–1999. Some participants were recruited through the general population whereas others were recruited through the healthcare system. These programs were designed to be enrichment programs featuring classroom and outdoor environments for ecologically valid assessment, rather than intensive therapeutic interventions. All participants and their families underwent a rigorous, multi-step psychodiagnostic assessment process (see below), after which 140 girls with ADHD and 88 age- and ethnicity-matched comparison girls were selected to participate in the childhood program (Wave 1; *M*_*age*_ = 9.6 years, range = 6–12 years).

Following recruitment, all participants were screened for ADHD regardless of if they had already had a pre-established diagnosis. To establish a baseline diagnosis of ADHD, we used the parent-administered Diagnostic Interview Schedule for Children, 4^th^ ed. (DISC-IV) [55] and SNAP rating scale [52], Hinshaw, [54] for the diagnostic algorithm). Comparison girls could not meet diagnostic criteria for ADHD on either measure. Some comparison girls met criteria for internalizing disorders (3.4%) or disruptive behavior disorders (6.8%) at baseline, yet our goal was not to match ADHD participants on comorbid conditions but instead to obtain a representative comparison group. Exclusion criteria included intellectual disability, pervasive developmental disorders, psychosis, overt neurological disorder, lack of English spoken at home, and medical problems preventing summer camp participation. The final sample included 228 girls with ADHD-Combined presentation (*n* = 93) and ADHD-Inattentive presentation (*n* = 47), plus an age- and ethnicity-matched comparison sample (*n* = 88). Participants were ethnically diverse (53% White, 27% African American, 11% Latina, 9% Asian American), reflecting the composition of the San Francisco Bay Area in the 1990’s. Family income was slightly higher than the median local household income in the mid-1990s, yet income and educational attainment of families were highly variable, ranging from professional families to those receiving public assistance. On average, parents reported being married and living together (65.8%) at the baseline assessment.

Participants were then assessed 5 (Wave 2; *M*_*age*_ = 14.2 years, range = 11–18; 92% retention [data not included from this wave in the present study]), 10 (Wave 3; *M*_*age*_ = 19.6 years, range = 17–24 years; 95% retention), and 16 (Wave 4; *M*_*age*_ = 25.6 years, range = 21–29 years; 93% retention) years later. Data collection included multi-domain, multi-informant assessments, performed in our clinic for most individuals; when necessary, we performed telephone interviews or home visits. We obtained informed consent from all participants (for initial waves: all legal guardians for minors (if age was below 18 years) and parents; for later waves: all participants and parents). Participants received monetary compensation. For additional information see Hinshaw et al. [31, 56], Owens et al. [57]. 

### Measures

#### Predictor variables

Predictor variables were measured during the baseline assessment at Wave 1 (childhood), with repeated assessment of several key measures at Wave 3 (late adolescence), to incorporate risk factors in both developmental periods.

*ADHD Symptom Severity: Swanson, Nolan, and Pelham rating scale, 4*^*th*^* Ed. (SNAP-IV;* Swanson, [58]*).* We measured severity of both hyperactivity/impulsivity (SNAP-HI) and inattentive (SNAP-IA) symptoms using an average of parent- and teacher-report (childhood) or parent- and self-report (late adolescence) on a dimensionalized checklist of these two respective symptom domains (9 items for each) to obtain multi-informant composite scores (SNAP-HI: α = 0.950; SNAP-IA: α = 0.968). For example, items included “…this child is forgetful in daily activities” and “…this child blurts out answers to questions before the questions have been completed.” The severity of each symptom was scored 0 (not at all) to 3 (very much). Thus, scores of both hyperactivity/impulsivity and inattention symptoms ranged from 0–27, with higher scores indicating more severe symptomology. The SNAP-IV is a widely used scale of ADHD symptom severity in both research and clinical settings (e.g., MTA Cooperative Group, [59]). It has good internal consistency and test–retest reliability [60].

*Executive Functioning: Rey Osterrieth Complex Figure (ROCF)* [61]. We measured executive functioning using the ROCF, a laboratory-based cognitive task requesting that an individual copy and later recall a complex image composed of 64 segments. The ROCF measures multiple domains of executive functioning such as planning, inhibitory control, attention to detail, working memory, and organization. It is often considered a more “global” measure of executive functioning [62]. We analyzed the Copy condition of this task, during which participants are timed as they view the stimulus figure and draw the figure on a blank piece of paper. For scoring, we used the Error Proportion Score (EPS; the ratio of number of errors divided by the total number of segments drawn), a well-validated method of scoring the ROCF, indexing efficiency [63]. In previous research with this sample, only the Copy condition (versus Delayed Recall condition) differentiated girls with ADHD from our comparison sample at baseline. The ROCF EPS showed the largest effect size (*d* = 0.90) out of all other EF measures in our battery (Hinshaw et al., [64]; Sami et al., [63]). As well, childhood EPS predicts later academic and occupational functioning in comparison to other EF measures Miller et al., [62]).

*Internalizing and Externalizing Symptoms: Child Behavior Checklist, Adult Self Report, and Adult Behavior Checklist (CBCL; ASR; ABCL)* [65, 66]*.* In childhood, we measured severity of internalizing (α = 0.89) and externalizing (α = 0.93) symptoms via parent-report on the Internalizing and Externalizing scales of the CBCL. In late adolescence, we averaged participant self-report on the Adult Self-Report (ASR) and parent-report on the Adult Behavior Checklist (ABCL) to obtain multi-informant composite scores. The ASR and ABCL are parallel versions of the CBCL for older individuals. We used *T*-scores (*M* = 50, *SD* = 10) as dimensional symptom measures, with scores above 60 indicating elevated/at-risk and scores above 70 indicating clinically significant symptoms. For example, items included: “…. your child feels worthless or inferior” (internalizing) “…your child gets in many fights” (externalizing). The CBCL, ASR, and ABCL have good–excellent validity, test–retest reliability, and internal consistency [66, 67].

*Parent Psychopathology: Beck Depression Inventory (BDI-I; BDI-II)* [68, 69]*.* We measured depressive symptoms of the primary caregiver (typically the mother) using self-report on the BDI-I at Wave 1 and the BDI-II at Wave 3. Mothers rated each of the 21 items on a 4-point severity scale. For example, items included a choice between “1.) I do not feel sad. 2.) I feel sad. 3.) I am sad all the time and I can’t snap out of it. 4.) I am so sad or unhappy I can’t stand it.” Total possible scores could range from 0–63, with higher scores indicating greater severity of depression. The BDI is a widely used and extensively validated self-report measure of depression in adults [70].

*Parenting Stress Due To Dysfunctional Interactions: Parenting Stress Index-Short Form (PSI-SF)* [71]*.* We measured stress-inducing dysfunctional parent–child interactions using the PSI-SF, a widely used self-report measure assessing stress experienced by parents related to their role as a parent. In particular, we used the 12-item Parental-Child Dysfunctional Interaction (PCDI) subscale which measures a parent’s perception of dysfunction in the parent–child relationship that contributes to the parent’s feelings of parental stress. Participants’ mothers rated each item on a scale from 1 (strongly agree) to 5 (strongly disagree). For example, items included: “My child does not like me or want to be close.” Higher scores indicated higher levels of maternal parenting stress. The PSI-SF has demonstrated good test-test reliability, internal consistency, and validity, with the reliability of the subscales ranging from 0.68 to 0.85 and the internal consistency ranging from 0.80 to 0.87 [72, 73]. In our sample, the internal consistency (Cronbach’s alpha) of the Parental-Child Dysfunctional Interaction subscale at Wave 1 and Wave 3 were 0.88 and 0.93 respectively.

*Cumulative childhood adversity: Adverse Childhood Experiences questionnaire (ACE)* [74]*.* We measured cumulative experiences of childhood adversity via retrospective report by on the ACE questionnaire at Wave 4, which assesses experiences of childhood abuse, neglect, and household dysfunction during the first 18 years of life. ACE scores ranged from 0–10, with higher scores indicating experiences of multiple types of childhood adversity. For example, items included: “Did you often or very often feel that no one in your family loved you or thought you were important or special?” The ACE questionnaire is a commonly used measure to assess for the cumulative effect of multiple forms of childhood adversity [75], and has good reliability and validity [76]—including at least moderate test–retest reliability of retrospective reports [77].

### Criterion variables

These were measured at Wave 4 (Young Adulthood).

*Borderline Personality Disorder Diagnosis.* A licensed clinical psychologist or a graduate student in clinical psychology, under close supervision, conducted a clinical interview with participants using the Structured Clinical Interview for DSM-IV-TR (SCID) [78] and the Borderline Personality Disorder (BPD) module of the SCID-II (SCID-II) [79]. The SCID-II is a semi-structured interview widely used in both research and clinical practice, with research indicating good to excellent inter-rater reliability [80]. A participant met criteria for a diagnosis of BPD if the clinician rated the participant at or above threshold on five of the nine symptom traits. A single dichotomous variable (0 or 1) reflected a BPD diagnosis.

*Borderline Personality Disorder Dimensional Features.* Because both diagnostic interview and self-report measures may yield optimal assessment of BPD [81], we also included a dimensional measure of BPD in order to assess and validate the categorical measure of BPD. For a large subset of the sample, a 15-item self-report scale was included, based on the BPD module of the Structured Clinical Interview for DSM-5 Axis II disorders (SCID-II) [82]. However, every participant did not complete this self-report measure, as it was added after data collection began. Additionally, some participants completed only interviews and did not return their packet of questionnaires including this measure. Each item of the measure is rated dichotomously (0 = No, 1 = Yes), so that the total possible score ranged from 0–15, with higher scores indicating more features of BPD. For example, items included: “Have you often become frantic when you thought that someone you really cared about was going to leave you?” This scale is consistent with DSM-5 BPD criteria, and has been used in several other studies, with satisfactory internal reliability (α = 0.81) [83, 84].

### Covariates

To ascertain whether domains of impairment were related specifically to BPD status, we added covariates empirically associated with BPD and associated predictors: (1) SES—a composite measure of parent report of family income and maternal education in childhood; (2) parent report of child’s race/ethnicity in childhood; and (3) participant age in young adulthood.

### Data analytic plan

Statistical analyses were performed with RStudio, version 1.2.1335. First, we computed descriptive statistics and zero-order correlations across potential predictors, background variables of interest, and young adult BPD (measured both categorically and dimensionally). Second, we conducted a series of (a) binary logistic regressions to test whether each theory-informed predictor independently predicted a young-adult diagnosis of BPD and (b) parallel linear regressions regarding dimensional features of BPD. We calculated effect sizes of Cohen’s *d* for the dichotomous criterion and *R*^2^ for the dimensional measure. Given the many initial predictors, we deployed the stringent criterion that a predictor be retained for subsequent analyses only if it displayed a medium (or larger) effect size in relation to the respective categorical or dimensional measure of BPD. For Cohen’s d, we considered effect sizes ≧ 0.2 as small, ≧ 0.5 as medium, and ≧ 0.8 as large; for *R*^2^, we considered ≧ 0.02 as small, ≧ 0.13 as medium, and ≧ 0.26 as large (Cohen, 1988). Third, we tested whether predictors meeting this criterion continued to do so when adjusting for sociodemographic covariates (baseline SES, participant race/ethnicity, and participant age), using (a) binary logistic regressions or (b) linear regressions, respectively.

We added predictors maintaining significance into separate models by developmental period (Model 1 = childhood; Model 2 = late adolescence), Given the small subset with a BPD diagnosis, we used Firth’s penalized likelihood method in binary logistic regressions to minimize bias introduced by several independent variables [85]. For exploratory moderator analyses, we conceptualized a moderator as a baseline factor that might reveal differential predictor-criterion associations at different levels of the putative moderator [86]. Our moderator of interest included baseline (Wave 1) socioeconomic status. Understanding that such analyses are non-hypothesis-driven, we placed interaction terms of the putative predictor x SES moderator at the third step of each significant predictor regression model.

## Results

### Descriptive analyses and correlations

A total of 19 participants met criteria for a diagnosis of BPD. Fourteen (74%) had received a childhood diagnosis of ADHD (χ^2^(3, *N* = 199) = 1.1, *p* = 0.3, *OR*: 1.31, CI: 0.79, 2.17), with a majority of them having received a childhood diagnosis of ADHD-C (58%).

Tables 1, 2, 3 and 4 present intercorrelations among key variables. Because maternal- and teacher-report of childhood hyperactivity/impulsivity (W1 SNAP-HI), inattention (W1 SNAP-IA), overt aggression (W1 CSBS), and relational aggression (W1 CSBS), plus maternal- and self-report of late adolescent hyperactivity/impulsivity (W3 SNAP-HI), inattention (W3 SNAP-IA), externalizing symptoms (W3 ASR/ABCL), and internalizing symptoms (W3 ASR/ABCL) were highly correlated, we averaged ratings across mother and teacher (childhood) and mother and self (early adulthood) to create a composite score for each domain.

**Table 1:** Descriptive statistics and correlations of childhood study variables with young adult BPD diagnosis

Variables of interest	No BPD (*n* = 182) M(*SD*)	BPD (*n* = 19) M(*SD*)	2	3	4	5	6	7	8	9	10	11	12
1 BPD Diagnosis (W4 SCID)	-	-	0.17^*a^	0.11^*^	0.22^*b^	0.09^*^	0.06^*^	0.04^*^	0.13^*^	0.10^*^	0.04^*^	-0.04^*^	0.32^*b^
2 Impulsivity (W1 SNAP-HI Mom/Teach Report)	8.51(7.56)	12.89(7.96)	-	0.77^b^	0.42^b^	0.78^b^	0.48^b^	0.69^b^	0.54^b^	0.59^b^	0.18^b^	0.34^b^	0.24^b^
3 Inattention (W1 SNAP-IA Mom/Teach Report)	12.52(8.76)	15.68(8.61)		-	0.28^b^	0.72^b^	0.59^b^	0.49^b^	0.49^b^	0.46^b^	0.11	0.43^b^	0.15^a^
4 Low Executive Functioning (W1 RCFT)	0.28(0.18)	0.42(0.22)			-	0.4^b^	0.22^b^	0.33^b^	0.21^b^	0.32^b^	-0.01	0.15^a^	0.11
5 Externalizing (W1 CBCL Mom Report)	57.41(13.6)	61.74(13.60)				-	0.7^b^	0.63^b^	0.49^b^	0.57^b^	0.2^b^	0.49^b^	0.22^b^
6 Internalizing (W1 CBCL Mom Report)	55.03(12.61)	57.53(11.10)					-	0.39^b^	0.3^b^	0.37^b^	0.28^b^	0.47^b^	0.27^b^
7 Overt Aggression (W1 CSBS Mom/Teach Report)	6.35(3.2)	6.82(2.72)						-	0.63^b^	0.64^b^	0.19^b^	0.26^b^	0.22^b^
8 Relational Aggression (W1 CSBS Mom/Teach Report)	13.76(4.93)	15.89(5.69)							-	0.38^b^	0.04	0.28^b^	0.18^a^
9 Negative Nominations (W1 Peer Report)	0.34(0.48)	0.51(0.67)								-	0.11	0.23^b^	0.2^b^
10 Maternal Psychopathology (W1 BDI)	5.33(5.20)	6.03(6.99)									-	0.25^b^	0.17^a^
11 Maternal Parenting Stress due to Dysfunctional Interactions (W1 PSI-PCDI)	2.05 (0.77)	1.95(0.67)										-	0.04
12 Cumulative History of Childhood Adversity (W4 ACEs)	1.74(1.76)	3.84(2.63)											-

*BPD*: Borderline Personality Disorder, *W4*: Wave 4, *SCID*: Structured Clinical Interview for DSM disorders, *W1*: Wave 1, *SNAP*: Swanson, Nolan and Pelham Questionnaire, *HI*: Hyperactivity/Impulsivity, *IA*: Inattention, *RCFT*: Rey Osterrieth Complex Figure Task, *CBCL*: Child Behavior Checklist, *CSBS*: Children’s Social Behavior Scale, *BDI*: Beck Depression Inventory, *PSI*: Parenting Stress Index, *PCDI*: Parent–Child Dysfunctional Interactions, *ACEs*: Adverse Childhood Experiences Scale

^*^Point Biserial Correlation

^a^ Correlation significant at 0.05 level

^b^ Correlation significant at 0.01 level

**Table 2:** Descriptive statistics and correlations of late adolescent study variables with young adult BPD diagnosis

Variables of interest	No BPD (*n* = 182) M(*SD*)	BPD (*n* = 19) M(*SD*)	2	3	4	5	6	7	8
1 BPD Diagnosis (W4 SCID)	-	-	0.40^*b^	0.38^*b^	0.01^*^	0.39^*b^	0.34^*b^	0.16^*a^	0.05^*^
2 Impulsivity (W3 SNAP-HI Mom/Self Report)	4.98(4.66)	12.29(7.86)	-	0.69^b^	0.21^b^	0.74^b^	0.56^b^	0.24^b^	0.32^b^
3 Inattention (W3 SNAP-IA Mom/Self Report)	8.65(6.52)	17.45(6.30)		-	0.11	0.69^b^	0.57^b^	0.28^b^	0.51^b^
4 Low Executive Functioning (W3 RCFT)	0.18(0.10)	1.18(0.10)			-	0.18^b^	0.12	0.01	0.18^a^
5 Externalizing Symptoms (W3 ASR/ABCL Mom/Self Report)	53.74(10.19)	68.11(10.89)				-	0.73^b^	0.26^b^	0.49^b^
6 Internalizing Symptoms (W3 ASR/ABCL Mom/Self Report)	52.46(10.67)	65.53(11.02)					-	0.34^b^	0.50^b^
7 Maternal Psychopathology (W3 BDI-II)	5.92(7.88)	10.41(12.12)						-	0.43^b^
8 Maternal Parenting Stress due to Dysfunctional Interactions (W3 PSI-PCDI)	2.04(0.85)	2.18(0.93)							-

*BPD*: Borderline Personality Disorder, *W4*: Wave 4, *SCID*: Structured Clinical Interview for DSM disorders, *W3*: Wave 3, *SNAP*: Swanson, Nolan and Pelham Questionnaire, *HI*: Hyperactivity/Impulsivity, *IA*: Inattention, *RCFT*: Rey Osterrieth Complex Figure Task, *ASR*: Adult Self-Report, *ABCL*: Adult Behavior Checklist, *BDI-II*: Beck Depression Inventory-II, *PSI*: Parenting Stress Index, *PCDI*: Parent–Child Dysfunctional Interactions

^*^Point Biserial Correlation

^a^ Correlation significant at 0.05 level

^b^ Correlation significant at 0.01 level

**Table 3:** Descriptive statistics and correlations of childhood study variables with young adult BPD features

Variables of interest	Mean	*SD*	*n*	2	3	4	5	6	7	8	9	10	11	12
1 Young Adult BPD Features	4.18	3.74	143	0.43^b^	0.29^b^	0.25^b^	0.36^b^	0.24^b^	0.38^b^	0.32^b^	0.35^b^	0.16	0.16	0.47^b^
2 Impulsivity (W1 SNAP-HI Mom/Teach Report)	9.30	7.69	143	-	0.73^b^	0.45^b^	0.78^b^	0.44^b^	0.7^b^	0.56^b^	0.66^b^	0.15	0.30^b^	0.23^b^
3 Inattention (W1 SNAP-IA Mom/Teach Report)	13.09	8.61	143		-	0.23^b^	0.66^b^	0.55^b^	0.49^b^	0.52^b^	0.47^b^	0.09	0.7^b^	0.14
4 Low Executive Functioning (W1 RCFT)	0.30	0.19	142			-	0.39^b^	0.21^a^	0.37^b^	0.24^b^	0.36^b^	-0.01	0.09	0.08
5 Externalizing (W1 CBCL Mom Report)	57.9	13.63	143				-	0.69^b^	0.68^b^	0.50^b^	0.61^b^	0.16	0.46^b^	0.22^b^
6 Internalizing (W1 CBCL Mom Report)	55.61	13.1	143					-	0.38^b^	0.28^b^	0.35^b^	0.25^b^	0.43^b^	0.33^b^
7 Overt Aggression (W1 CSBS Mom/Teach Report)	6.37	3.23	138						-	0.61^b^	0.73^b^	0.19^a^	0.27^b^	0.18^a^
8 Relational Aggression (W1 CSBS Mom/Teach Report)	14.13	4.87	134							-	0.45^b^	-0.05	0.29^b^	0.17
9 Negative Nominations (W1 Peer Report)	0.36	0.47	143								-	0.08	0.22^b^	0.15
10 Maternal Psychopathology (W1 BDI)	5.21	5.01	138									-	0.19^b^	0.22^b^
11 Maternal Parenting Stress due to Dysfunctional Interactions (W1 PSI-PCDI)	1.99	0.73	142										-	0.04
12 Cumulative History of Childhood Adversity (W4 ACEs)	1.22	0.42	144											-

*BPD*: Borderline Personality Disorder, *W1*: Wave 1, *SNAP*: Swanson, Nolan and Pelham Questionnaire, *HI*: Hyperactivity/Impulsivity, *IA*: Inattention, *RCFT*: Rey Osterrieth Complex Figure Task, *CBCL*: Child Behavior Checklist, *CSBS*: Children’s Social Behavior Scale, *BDI*: Beck Depression Inventory, *PSI*: Parenting Stress Index, *PCDI*: Parent–Child Dysfunctional Interactions, *ACEs*: Adverse Childhood Experiences Scale, *W1*: Wave 1, *W4*: Wave 4

^a^ Correlation significant at 0.05 level

^b^ Correlation significant at 0.01 level

**Table 4:** Descriptive statistics and correlations of late adolescent study variables with young adult BPD features

Variables of interest	Mean	*SD*	*n*	2	3	4	5	6	7	8
1 Young Adult BPD Features	4.18	3.74	143	0.52^b^	0.43^b^	0.20^a^	0.63^b^	0.57^b^	0.25^b^	0.23^a^
2 Impulsivity (W3 SNAP-HI Mom/Self Report)	5.84	5.38	136	-	0.62^b^	0.30^b^	0.69^b^	0.52^b^	0.25^b^	0.20^a^
3 Inattention (W3 SNAP-IA Mom/Self Report)	10.11	6.91	137		-	0.17^a^	0.63^b^	0.55^b^	0.24^b^	0.43^b^
4 Low Executive Functioning (W3 RCFT)	0.18	0.09	135			-	0.25^b^	0.18^a^	0.08	0.17
5 Externalizing Symptoms (W3 ASR/ABCL Mom/Self Report)	55.61	10.84	137				-	0.7^b^	0.28^b^	0.42^b^
6 Internalizing Symptoms (W3 ASR/ABCL Mom/Self Report)	54.93	11.03	137					-	0.44^b^	0.51^b^
7 Maternal Psychopathology (W3 BDI-II)	6.36	9.24	115						-	0.49^b^
8 Maternal Parenting Stress due to Dysfunctional Interactions (W3 PSI-PCDI)	2.03	0.83	114							-

*BPD*: Borderline Personality Disorder, *W3*: Wave 3, *SNAP*: Swanson, Nolan and Pelham Questionnaire, *HI*: Hyperactivity/Impulsivity, *IA*: Inattention, *RCFT*: Rey Osterrieth Complex Figure Task, *ASR*: Adult Self-Report, *ABCL*: Adult Behavior Checklist, *BDI-II*: Beck Depression Inventory-II, *PSI*: Parenting Stress Index, *PCDI*: Parent–Child Dysfunctional Interactions

^a^ Correlation significant at 0.05 level

^b^ Correlation significant at 0.01 level

For young-adult categorical BPD diagnoses, significant childhood point biserial correlates included hyperactivity/impulsivity (W1 SNAP-HI; *r*_*pb*_ = 0.17, *p* < 0.05), low executive functioning (W1 ROCF; *r*_*pb*_ = 0.22, *p* < 0.01), and a history of overall adversity (W4 ACEs; *r*_*pb*_ = 0.32, *p* < 0.01) (see Table 1). Significant late-adolescent point biserial correlates included hyperactivity/impulsivity (W3 SNAP-HI; *r*_*pb*_ = 0.40, *p* < 0.01), inattention (W3 SNAP-IA; *r*_*pb*_ = 0.38, *p* < 0.01), externalizing symptoms (W3 ASR/ABCL; *r*_*pb*_ = 0.39, *p* < 0.01), internalizing symptoms (W3 ASR/ABCL*; r*_*pb*_ = 0.34, *p* < 0.01), and maternal psychopathology (W3 BDI-II (*r*_*pb*_ = 0.16, *p* < 0.05) (see Table 2).

Regarding young-adult dimensionally scored features of BPD, childhood hyperactivity/impulsivity (W1 SNAP-HI; *r* = 0.43, *p* < 0.01), childhood inattention (W1 SNAP-IA; *r* = 0.29, *p* < 0.01), low executive functioning (W1 ROCF; *r* = 0.25, *p* < 0.01), externalizing symptoms (W1 CBCL; *r* = 0.36, *p* < 0.01), internalizing symptoms (W1 CBCL; *r* = 0.24, *p* < 0.01), overt aggression (W1 CSBS; *r* = 0.38, *p* < 0.01), relational aggression (W1 CSBS; *r* = 0.32, *p* < 0.01), negative peer nominations (W1 Peer Report; *r* = 0.35, *p* < 0.01), and a cumulative history of childhood adversity (W4 ACEs; *r* = 0.47, *p* < 0.01) were significant correlates. Late adolescent hyperactivity/impulsivity (W3 SNAP-HI; *r* = 0.52, *p* < 0.01), inattention (W3 SNAP-IA; *r* = 0.43, *p* < 0.01), low executive functioning (W3 ROCF;; *r* = 0.20, *p* < 0.05), externalizing symptoms (W3 ASR/ABCL; *r* = 0.63, *p* < 0.01), internalizing symptoms (W3 ASR/ABCL*; r* = 0.57, *p* < 0.01), maternal psychopathology (W3 BDI-II; *r* = 0.25, *p* < 0.01), and maternal parenting stress due to dysfunctional interactions (W3 PSI-PCDI; *r* = 0.23, *p* < 0.05) were all significantly correlated with young adult BPD features (Table 4).

### Predictors of young adult BPD diagnosis

In the binary logistic regressions with Firth’s penalized likelihood method, conducted to assess independent predictors of the dichotomous outcome of meeting (vs. not meeting) diagnostic criteria for BPD in young adulthood, we initially tested whether each predictor of interest was significantly associated with BPD, followed by inclusion of (a) covariates and (b) other significant predictor variables according to developmental period (Table 5).

**Table 5:** Predictors of Risk for Young Adult BPD Diagnosis and Features

	**Young Adult BPD Diagnosis (*n* = 201)**			**Young Adult BPD Features (*n* = 143)**		
	***p***	**Cohen’s *d***	***p* with covariates**^**1**^	***p***	***R***^**2**^	***p* with covariates**^**1**^
**Childhood Predictors**						
Impulsivity (W1 SNAP-HI Mom/Teach Report)	0.022*	0.58	0.094	0.000***	0.182	0.000***
Inattention (W1 SNAP-IA Mom/Teach Report)	0.15	0.36	0.244	0.000***	0.079	0.000***
Low Executive Functioning (W1 RCFT)	0.004**	0.76	0.031*	0.004**	0.054	0.293
Externalizing (W1 CBCL Mom Report)	0.20	0.32	0.468	0.000***	0.121	0.000***
Internalizing (W1 CBCL Mom Report)	0.41	0.20	0.789	0.004**	0.051	0.025*
Overt Aggression (W1 CSBS Mom/Teach Report)	0.46	0.15	0.958	0.000***	0.137	0.000***
Relational Aggression (W1 CSBS Mom/Teach Report)	0.078	0.43	0.089	0.000***	0.094	0.000***
Negative Nominations (W1 Peer Report)	0.13	0.35	0.462	0.000***	0.114	0.000***
Maternal Psychopathology (W1 BDI)	0.48	0.13	0.891	0.045*	0.020	0.205
Maternal Parenting Stress due to Dysfunctional Interactions (W1 PSI-PCDI)	0.64	0.13	0.633	0.058	0.019	0.017*
**Childhood-Adolescent Predictors (Retrospectively Reported)**						
Cumulative History of Childhood Adversity (W4 ACEs)	0.000***	1.14	0.005**	0.000***	0.213	0.000***
**Late Adolescence Predictors**						
Impulsivity (W3 SNAP-HI Mom/Self Report)	0.000***	1.45	0.000***	0.000***	0.265	0.000***
Inattention (W3 SNAP-IA Mom/Self Report)	0.000***	1.36	0.000***	0.000***	0.177	0.000***
Low Executive Functioning (W3 RCFT)	0.80	0.04	0.994	0.023*	0.033	0.058
Externalizing Symptoms (W3 ASR/ABCL Mom/Self Report)	0.000***	1.41	0.000***	0.000***	0.398	0.000***
Internalizing Symptoms (W3 ASR/ABCL Mom/Self Report)	0.000***	1.23	0.000***	0.000***	0.317	0.000***
Maternal Psychopathology (W3 BDI-II)	0.046*	0.54	0.093	0.008**	0.053	0.010*
Maternal Parenting Stress due to Dysfunctional Interactions (W3 PSI-PCDI)	0.496	0.17	0.46	0.016*	0.043	0.003**

*BPD*: Borderline Personality Disorder, *W1*: Wave 1, *SNAP*: Swanson, Nolan and Pelham Questionnaire, *HI*: Hyperactivity/Impulsivity, *IA*: Inattention, *RCFT*: Rey Osterrieth Complex Figure Task, *CBCL*: Child Behavior Checklist, *CSBS*: Children’s Social Behavior Scale, *BDI*: Beck Depression Inventory, *PSI*: Parenting Stress Index, *PCDI*: Parent–Child Dysfunctional Interactions, *ACEs*: Adverse Childhood Experiences Scale, *W4*: Wave 4, W3: Wave 3, *ASR*: Adult Self-Report, *ABCL*: Adult Behavior Checklist, *BDI-II*: Beck Depression Inventory-II

^1^Covariates: Race, Family SES at Wave 1; Participant age at Wave 4

^*^Correlation significant at 0.05 level

^**^Correlation significant at 0.01 level

^***^Correlation significant at 0.001 level

Among childhood predictors, hyperactivity/impulsivity (*p* < 0.05; *d* = 0.58) and low executive functioning (*p* < 0.01; *d* = 0.76) each predicted BPD diagnostic status in young adulthood, but only low executive functioning maintained significance after adjusting for covariates (*p* < 0.05). As well, the childhood ACE score was a significant predictor, even with adjustment for covariates (*p* < 0.001; *d* = 1.14). Regarding for late-adolescent predictors, hyperactivity/impulsivity (*p* < 0.001; *d* = 1.45), inattention (*p* < 0.001; *d* = 1.36), externalizing (*p* < 0.001; *d* = 1.41), and internalizing (*p* < 0.001; *d* = 1.23) symptoms each predicted young adult BPD, adjusting for covariates. Maternal psychopathology did not survive inclusion of covariates (*p* = 0.093).

Finally, we entered all predictors with a medium or larger effect size (Cohen’s d ≧ 0.5) that had maintained significance after inclusion of covariates into models divided by developmental period. In childhood, low executive functioning (*p* = 0.012) and the ACE score maintained significance (*p* = 0.003). In the late-adolescent predictor model, only inattentive symptoms maintained marginal significance (*p* = 0.059), but hyperactivity/impulsivity (*p* > 0.05), internalizing symptoms (*p* > 0.05), and externalizing symptoms (*p* > 0.05) did not.

### Predictors: Young adult dimensional BPD features

Via linear regressions, childhood hyperactivity/impulsivity (*p* < 0.001; *R*^2^ = 0.182), inattention (*p* < 0.001; *R*^2^ = 0.079), low executive functioning (*p* = 0.004; *R*^2^ = 0.054), externalizing symptoms (*p* < 0.001; *R*^2^ = 0.121), internalizing symptoms (*p* = 0.004; *R*^2^ = 0.051), overt aggression (*p* < 0.001; *R*^2^ = 0.137), relational aggression (*p* < 0.001; *R*^2^ = 0.094), negative peer nominations (*p* < 0.001; *R*^2^ = 0.114), and maternal psychopathology (*p* = 0.045; *R*^2^ = 0.020) independently predicted young-adult features of BPD. Of these, only childhood hyperactivity/impulsivity (*p* < 0.001), inattention (*p* < 0.001), externalizing symptoms (*p* < 0.001), internalizing symptoms (*p* < 0.05), overt aggression (*p* < 0.001), relational aggression (*p* < 0.001), and negative peer nominations (*p* < 0.001) maintained significance after adjusting for covariates. Maternal parenting stress due to dysfunctional interactions became significant after adjusting for covariates (*p* < 0.05). The ACE score significantly predicted young adult BPD features (*p* < 0.001; *R*^2^ = 0.213), even after adjusting for covariates (*p* < 0.001).

For late-adolescent predictors, hyperactivity/impulsivity (*p* < 0.001; *R*^2^ = 0.265), inattention (*p* < 0.001; *R*^2^ = 0.177), low executive functioning (*p* < 0.05; *R*^2^ = 0.033), externalizing symptoms (*p* < 0.001; *R*^2^ = 0.398), internalizing symptoms (*p* < 0.001; *R*^2^ = 0.317), maternal psychopathology (*p* < 0.01; *R*^2^ = 0.053), and maternal parenting stress due to dysfunctional interactions (*p* < 0.05; *R*^2^ = 0.043) each independently predicted features of BPD in young adulthood. Of these, hyperactivity/impulsivity (*p* < 0.001), inattention (*p* < 0.001), externalizing symptoms (*p* < 0.001), internalizing symptoms (*p* < 0.001), maternal psychopathology (*p* < 0.05), and maternal parenting stress due to dysfunctional interactions (*p* < 0.01) maintained significance after adjusting for covariates. Low executive functioning did not.

Finally, we entered predictors with a medium (or above) effect size (*R*^2^ ≧ 0.13)—that had maintained significance after inclusion of covariates—into separate models by developmental period. In childhood, only childhood hyperactivity/impulsivity (*p* < 0.01) and the ACE score maintained significance (*p* < 0.001), but overt aggression did not (*p* > 0.05). As for late-adolescent predictors, only externalizing (*p* < 0.001) and internalizing symptoms (*p* < 0.01) maintained significance—but not hyperactivity/impulsivity (*p* > 0.05) or inattention (*p* > 0.05).

### Exploratory moderator analyses

Regarding categorical young adult BPD diagnosis, no predictor x moderator interactions emerged as statistically significant. For young adult dimensional BPD features, only an interaction between (a) low childhood executive functioning (predictor) and (b) low childhood socioeconomic status (moderator) (W1 SES; Δ*R*^2^ = 0.022, *p* < 0.05) emerged as statistically significant. Here, it was the combination of low executive functioning and low baseline SES that predicted higher levels of BPD dimensional features.

## Discussion

Leveraging a well-characterized longitudinal female sample with and without carefully diagnosed childhood ADHD, we examined theory-informed predictors of young adult BPD—considered both categorically and dimensionally—from childhood and late-adolescent timespans. Although we emphasize caution regarding interpretation of findings due to our small sample size, this investigation extends research from our laboratory on developmental predictors of self-harm behaviors [87] to include borderline personality disorder as a criterion measure. We note that individuals with BPD—a condition characterized by intense and pervasive dysregulation of emotion, behavior, cognition, and relationships—may or may not engage in self-harm.

First, regarding our categorical measure of BPD, using binary logistic regressions with correction for small sample size, we found that—as hypothesized—a cumulative history of childhood adversity, as measured by the ACE score, predicted BPD diagnosis. Low EF in childhood was also a significant predictor, even after adjusting for ACE scores and demographic covariates. Regarding late-adolescent predictors, hyperactivity/impulsivity, inattention, internalizing, and externalizing symptoms each independently predicted young adult BPD diagnosis after adjusting for covariates, but maternal depression did not. In stringent analyses accounting for all independently significant late adolescent predictors, only symptoms of inattention were independently (albeit marginally) related to young adult BPD diagnosis.

Second, with respect to our dimensional measure of BPD features, we found—consistent with hypotheses—that both childhood hyperactivity/impulsivity and a cumulative history of childhood adversity from the ACE score predicted young adult BPD features, with adjustment for covariates. Furthermore, childhood inattention, externalizing symptoms, internalizing symptoms, overt aggression, relational aggression, negative peer nominations, and maternal parenting stress due to dysfunctional interactions also independently predicted young adult BPD features after adjusting for covariates. Yet in the final model, including all childhood predictors with a medium (or larger) effect size that had survived covariates, only childhood hyperactivity/impulsivity and the ACE score maintained significance. As for late-adolescent predictors of the dimensional outcome, hyperactivity/impulsivity, inattention, externalizing symptoms, internalizing symptoms, maternal psychopathology, and maternal parenting stress due to dysfunctional interactions maintained significance after adjusting for covariates, but low executive functioning did not. In the final analysis, adding all surviving predictors in the same model, only late-adolescent externalizing and internalizing symptoms maintained significance. Finally, as for exploratory moderator analyses, we found an interaction between low childhood executive functioning and low socioeconomic status at baseline was significant, suggesting that socioeconomic disadvantage may compound the predictive effects of low executive functioning with respect to later BPD dimensional scores.

Overall, the child and adolescent predictors of later BPD are largely consistent with those from previous investigations [8, 10, 18, 25, 26, 43], emerging here from a carefully controlled prospective investigation. Regarding ADHD symptoms, almost 75% of women who met criteria for BPD in young adulthood had diagnoses of childhood ADHD, most often characterized by high levels of impulsivity (ADHD-C). This finding is consistent with both cross-sectional and longitudinal research, as well as theoretical models of the developmental course of individuals with high levels of early impulsivity, related to BPD as an end-point [11].

For ADHD dimensions, our findings add to the limited number of studies examining hyperactive, impulsive, and inattentive symptoms and their severity across development, especially beginning in childhood [8, 25–27]. That hyperactivity/impulsivity in childhood and adolescence did not significantly predict later categorical BPD diagnosis was unexpected and may relate to our small sample size. Yet regarding our dimensional measure, when adjusting for covariates and other predictors, hyperactivity/impulsivity in childhood did significantly predict later BPD features. This finding is consistent with the only two other known studies to our knowledge that have examined prospective associations between childhood impulsivity and later BPD [26, 27]. Two studies have found prospective prediction from both adolescent hyperactivity/impulsivity and inattention to later BPD [8, 25]—along this line, note our marginally significant prediction of categorical BPD from late-adolescent inattentive symptoms: In both Carlson et al. [25] and a recent machine learning study of 128 variables related to risk for BPD, inattention in adolescence emerged as an important predictor [8]. Each dimension of ADHD appears to play an important role in the development of BPD symptoms.

The finding linking low EF in childhood to young adult BPD diagnosis is also consistent with previous (yet limited) research. In the only known prospective longitudinal study to date examining childhood EF as predictive of later BPD, a composite measure of EF at age 5 predicted BPD symptoms at age 12 [26]. When we examined low childhood EF and BPD dimensional features, our findings were not significant. Still, moderator analyses revealed that girls with both low EF *and* low socioeconomic status were at especially risk for high levels of BPD features. Perhaps difficulties in low EF are related to high or clinically significant BPD in the context of socioeconomic disadvantage.

Regarding internalizing and externalizing symptoms, we found that high levels of each were related to later BPD features after adjusting for other important predictors—but not when we measured BPD categorically. Furthermore, multiple forms of aggression in childhood including both overt and relational aggression, plus negative peer nominations, predicted young adult BPD features, but these findings did not maintain significance in the presence of other important predictors. Thus, symptoms of aggression and peer preference are important in childhood as risk factors for later BPD, yet other factors—childhood hyperactivity/impulsivity and trauma-remain statistically superior. Indeed, there was substantial overlap in our measures of aggression, negative peer nomination, hyperactivity/impulsivity, and broadband externalizing symptoms. Overall, our key findings replicate those from Stepp et al. [43], who showed that adolescent internalizing and externalizing symptoms predict adult BPD (for additional research, see Belsky et al., [26]; and Geselowitz et al., [10]). Adolescence appears to be a particularly sensitive period during which vulnerability for the development of severe and pervasive dysregulation across the lifespan may be realized [44].

Although maternal psychopathology—plus both child and late-adolescent maternal parenting stress due to dysfunctional interactions—were each independently associated with young adult BPD features after adjusting for covariates, their effect sizes were small. Any implications require replication. Findings from other research indicate that parental invalidation and negative parenting practices may well be stronger predictors [53].

Consistent with a large body of research linking a history of childhood adversity/trauma with later BPD [88], we found that a cumulative history of childhood adversity, measured by the ACE scale, was a crucial predictor of both young-adult BPD diagnosis and dimensional features. This measure of cumulative history of childhood adversity is retrospective—and may therefore be better characterized as a subjective experience of childhood trauma rather than objective experiences of childhood trauma. These findings are consistent recent data finding that risk of psychopathology is high among individuals with subjective reports of childhood maltreatment regardless of whether these experiences were validated by objective measures [89]. As well, the ACE measure we used constitutes the gold standard in the field.

Our results support theories that transactions between dispositional and environmental factors over time can lead to a cycle of dysregulation of emotion, behavior, and cognition as well as difficult interpersonal relationships [1, 11]. Indeed, findings support Linehan’s Biosocial Theory plus recent developmental models of females with ADHD [11]. That is, behaviorally expressed impulsivity may be a risk factor for a range of outcomes, including BPD. As development progresses, children with trait impulsivity may experience childhood trauma—linked with, for example, intergenerational trauma, maladaptive parenting practices (especially in relation to the child’s impulsive behavior), and/or parents’ own behavioral impulsivity—which may then transactionally escalate the development of BPD. The original impulsivity may, via heterotypic continuity, come to be expressed as a combination of internalizing *and* externalizing dimensions, leading to BPD [11, 90]—a suggestion requiring further empirical investigation. Future research should include prospective temporal assessment of these domains, as well as other environmental mediators (e.g., peer relationship influences, substance use) and valid measures of behavioral parental invalidation, to assess multi-factor etiological influences.

### Clinical implications

Although our sample size is too small to draw definitive clinical recommendations, we provide several ideas for possible clinical and public health implications, emphasizing caution in interpretation of results related to study limitations (see below for more detail in this regard). First, findings highlight the longstanding effects of early experiences of adversity and trauma. Prevention of these childhood experiences, especially through public health initiatives, cannot be overemphasized. Second, our results reveal the importance including global EF deficits in childhood as indicators of risk for BPD, in addition to the focus on childhood impulsivity. These findings have implications for guiding early clinical assessment and intervention (e.g., through early EF skills training) to prevent later BPD. In short, we highlight the need for interventions *before* the adolescent period, which appears to be an especially sensitive time of risk [11].

Children with histories of adversity/trauma and/or deficits in EF could receive interventions targeting emotion dysregulation, a mechanism linked to the development of BPD, such as Dialectical Behavior Therapy for Children (DBT-C) [91], Parent–Child Interaction Therapy (PCIT) [92], or creative combinations of these therapies [93]. Widespread assessment of early risk factors to identify individuals at risk remains a challenge. We also recommend that evidence-based treatments for severe emotion dysregulation (i.e., DBT) include remediation of EF deficits.

### Limitations and future directions

Our study has several important limitations. First, our sample size is small for the categorical BPD variable, with only 19 females meeting diagnostic criteria for BPD in young adulthood, clearly limiting statistical power. Note that we used Firth’s penalized likelihood method to statistically account for our small sample [94]. Furthermore, only a subset of our sample completed the self-report dimensional BPD measure. We emphasize the need for replication and cautious interpretation of findings. Second, we did not have symptom-level data available for our categorical measure of BPD, preventing us from evaluating clinician-assessed dimensions of BPD symptoms. Future research would benefit from examining dimensional severity of BPD symptoms, as well as specific traits, some of which have recently been linked to increased risk for a suicide attempt [95]. Third, several measures—including those of BPD features, cumulative trauma history, and ADHD (in part) were self-reported—and may thus be subject to bias. Fourth, we were not able to peform mediator analyses and therefore cannot add to the literature on potential “driving” mechanisms between childhood and adulthood (e.g., emotion dysregulation). Fifth, we did not separate predictor symptom domains of hyperactivity vs. impulsivity, as psychometrics are superior when using the full 9-item Hyperactivity/Impulsivity scale. As well, this measure is more consistent with the DSM’s layout of symptoms. Although we support the separation of theses symptoms in future research—see the excellent national analysis by Tiger et al. [27]—we elected to include the full 9-item scale. Sixth, our measure of externalizing symptoms in late adolescence (ASR, ABCL) included measures of aggression, but given our multiple testing, we did not examine aggression per se during this developmental window. Future research would benefit from examining aggressive symptoms across development, given empirical research and theory linking high levels aggression and peer problems with later BPD [11, 18]. Seventh, there is controversy over whether the Rey-Osterrieth Complex Figure Test captures meaningful variance in executive functioning [96]. It could be that this measure is a better index of visual-motor integration and overall neuropsychological functioning than of executive functioning [97, 98]. Future research should investigate different domains of neuropsychological functioning and BPD development. Eighth, there is definitional overlap between ADHD and BPD, given that both are characterized by impulsivity, which could account for some of the present results. Finally, a key limitation is the timing of our BPD measure—we measured BPD only during young adulthood, but some participants may already have met criteria for BPD in adolescence.

Still, key strengths include a carefully diagnosed, ethnically and socioeconomically diverse sample of females; emphasis on multi-domain and multi-informant measures; high sample retention; and a prospective (and ongoing) longitudinal design. Moreover, we included stringent use of covariates and statistical penalization. Finally, we examined multiple domains of risk for BPD simultaneously and included several measures of BPD symptomology.

## Conclusion

The current findings add to existing research on developmental pathways to BPD, especially among females with ADHD. Future directions should include replication, further examination of dimensions of both ADHD and EF, and distinct types of traumatic life events across development [99] as related to later BPD. Sensitive measures of early emotional invalidation are also necessary [53].

## Data Availability

The datasets used and/or analyzed during the current study are available from the corresponding author on reasonable request.

## Abbreviations

ACEs: Adverse childhood experiences scale
ADHD: Attention-deficit/hyperactivity disorder
ADHD-C: Attention-deficit/hyperactivity disorder, combined presentation
ADHD-IA: Attention-deficit/hyperactivity disorder, inattentive presentation
BDI: Beck depression inventory
BPD: Borderline personality disorder
CBCL: Child behavior checklist
CSBS: Children’s social behavior scale
EF: Executive functioning
HI: Hyperactivity/impulsivity
IA: Inattention
NSSI: Nonsuicidal self-injury
PSI: Parenting stress index
PCDI: Parent–child dysfunctional interactions
RCFT: Rey osterrieth complex figure task
SCID: Structured clinical interview for DSM disorders
SNAP: Swanson, Nolan, and Pelham questionnaire
W1: Wave 1
W3: Wave 3
W4: Wave 4
